# Opportunities and Challenges of Predictive Approaches for Harnessing the Potential of Genetic Resources

**DOI:** 10.3389/fpls.2021.674036

**Published:** 2021-07-01

**Authors:** Johannes W. R. Martini, Terence L. Molnar, José Crossa, Sarah J. Hearne, Kevin V. Pixley

**Affiliations:** International Maize and Wheat Improvement Center, Texcoco, Mexico

**Keywords:** genetic resources, germplasm bank collections, predictive breeding, genomic selection, pre-breeding

## Introduction

Favorable variation from genetic resources is anticipated to play a key role in the adaptation of crops to the increasingly unfavorable production conditions resulting from climate change (FAO, 2015). Weather extremes lead to more frequent occurrences of abiotic stress and facilitate the emergence and spread of diseases. While there is no doubt that alleles and haplotypes offered by accessions from germplasm banks are of enormous value, the integration of beneficial alleles into elite material poses three major challenges:

1. the identification of promising germplasm bank accessions,
2. the separation of beneficial major effect alleles from undesired linkage drag,
3. the repackaging of polygenic variation into elite and adapted materials.

Identifying promising germplasm bank accessions, which may offer single alleles with major effects or beneficial quantitative variation, often resembles looking for a needle in a haystack. In practice, it is almost never possible to phenotype a large portion of the available germplasm due to high costs, challenges with adaptation, restricted facility resources and time pressure. An informed prescreening of the available accessions will be necessary.

Moreover, when accessions with putative alleles for desired traits are identified, the mission is not yet accomplished, since the beneficial variation must be integrated into elite germplasm. In the case of a simple genetic architecture such as an identified major effect gene, the novel allele can be introgressed by marker assisted backcrossing (MABC) or can be approached by gene editing. However, preceding discovery research is required to identify the genetic variation associated with the phenotypic variation. In particular, gene editing requires very precise information on the causative variation. The availability of a trait-associated marker, which may be sufficient for an application in MABC, may be insufficient for a gene editing approach. This research is resource and time consuming and carries the inherent risk of unsuccessful validation experiments due an altered effect of the allele when in combination with the genetic background of elite material.

When dealing with quantitative variation, dedicated mapping experiments are not required. However, it is more difficult to bring quantitative variation into an elite background and have a product acceptable to breeders. Landraces carry many deleterious and inferior alleles which can quickly disrupt the positive linkage blocks painstakingly constructed by breeders over decades. Diminished agronomic performance makes the breeding community reluctant to include such germplasm in their elite breeding programs.

Prediction approaches can help the effective use of genetic resources in two ways. First, predictions can identify the most promising candidate accessions for a certain trait, thus restricting the number of accessions to evaluate in experiments (Yu et al., 2016). Second, predictions can accelerate the pre-breeding (or “germplasm enhancement”) process by helping to target the desired alleles for transfer to an elite germplasm background, saving resources and time.

In this commentary, we summarize some activities related to predictive breeding in the context of genetic resources conducted at the International Maize and Wheat Improvement Center (CIMMYT). We then discuss differences between predictive breeding approaches for genetic resources and genomic selection for elite breeding programs. We propose that research on predictive methods for genetic resources should explore approaches which are “enriched” by external information; for example, knowledge of molecular biological mechanisms, or accession “passport” data that provides information on the environmental conditions in which the accession was originally cultivated. Passport data comprising latitude, longitude, and altitude are fundamental initial information for each accession stored in the bank. The inclusion of external information may increase the power of predictive breeding approaches, especially in the context of harnessing genetic resources.

## Predictive Breeding for Genetic Resources at Cimmyt

### Genotyping of Accessions of CIMMYT's Germplasm Bank

CIMMYT has genotyped most of its maize and wheat collections as part of the Seeds of Discovery Project (SEED). For maize, more than 98% of the CIMMYT and IITA (International Institute of Tropical Agriculture) maize collection have been genotyped. For wheat, 37 and 66%, respectively, of the CIMMYT and ICARDA (International Center for Agricultural Research in the Dry Areas) wheat collection have been genotyped (Sansaloni et al., 2020). The smaller percentages for wheat, compared to maize, are due to the larger size and differing composition of the combined collections. CIMMYT's germplasm bank has ~28,000 maize, but more than 140,000 wheat accessions. The available genotypic data provides a solid foundation for prediction approaches for screening the collections more systematically.

### Genetic Resources for Breeding for Maize Lethal Necrosis Resistance

A recent example of the successful use of germplasm bank material in response to an emerging threat was the development of germplasm tolerant to Maize Lethal Necrosis (MLN). Thirteen out of 1000 screened landraces were identified as showing low susceptibility to Maize Chlorotic Mottle Virus (MCMV), the major causal component of MLN disease (for a review on CIMMYT's activities related to MLN, see Boddupalli et al., 2020). The pre-screening in this study was based on geographical distribution, racial structure, and genomic distance data calculated as described in Franco-Duran et al. (2019). The performance of the developed inbred lines in hybrid combinations is currently tested, in particular under MLN pressure.

### Prediction of Wheat Landraces Accessions

For wheat, Crossa et al. (2016) considered genomic prediction on a large set of Mexican (~8,400) and Iranian (~2,400) bank accessions for several traits including thousand-kernel weight, grain hardness, grain protein, and plant height. The predictive abilities obtained were mostly between 0.39 and 0.68, when using 20% of the data as training set (Crossa et al., 2016, Table 2). An exception was plant height for the Iranian landraces, which showed a predictive ability of only 0.17. These results indicated that genomic prediction has a potential for (1) fast screening of the whole GB for different traits, and (2) a rapid and efficient pre-breeding method for introgression useful alleles (and haplotypes) into advance breeding lines while not eroding genetic diversity.

### Association Studies With Environmental Covariates as Phenotype

A novel approach to use “passport” data of accessions is “environmental genome-wide association studies” (environmental GWAS or EnvGWAS). This approach treats environmental variables of the sites where accessions were collected as phenotypes, and combines this information with genotypic data for the accessions in an association study. The objective is to identify genetic variation which is associated with the adaptation to certain environmental conditions (Lasky et al., 2015; Romero Navarro et al., 2017; Gates et al., 2019). Though this approach conceptually could lead to high false positive rates due spatial distribution impacting phylogeny and environmental variables, this problem can be controlled, as in standard GWAS, by introducing a random polygenetic effect with the genomic relationship as covariance (Yang et al., 2014). Proof of concept work in drought using collection site precipitation data has demonstrated the power of EnvGWAS to detect variants of potential interest in maize landraces (Gates et al., 2019). Validation of the role of these variants in drought response, conducted through independent in silico analysis of transcriptome data and analysis of phenotypic data, has confirmed the value of EnvGWAS for identifying variants and in turn landraces containing variants for further analysis and use in breeding.

## Differences Between Predictive Approaches in the Context of Genetic Resources and Genomic Selection in an Elite Germplasm Pool

Although we have witnessed promising results for both maize and wheat, we see conceptual limitations of standard genomic prediction methods when looking for ***novel*** beneficial alleles. Standard prediction approaches predict from a training to a prediction set and can only predict the effect of new combinations of already known segments (Meuwissen et al., 2001). Indeed, this is also the major application of genomic selection in an elite breeding pipeline where most alleles have already been sampled in different combinations. In this situation, one aims at ***recombining*** the positive alleles which have already been observed. This differs fundamentally from a prediction where the objective is to find novel beneficial variation. Therefore, when screening for novel diversity which is not present in the training set, we see the main value of the prediction in its indirect information: a strong accumulation of beneficial alleles that are already present in the training set may be a result of selection pressure in the accession's history. Thus, the probability of finding additional novel alleles for the trait of interest may be increased.

### Approaches to Incorporate External Information

To address this conceptual discrepancy between the nature of statistical prediction and the objective of predicting novel diversity, and to go beyond the indirect information provided by a standard genomic selection as described above, we believe different sources of information need to be combined with genotypic data. Examples may be passport data as in EnvGWAS, gene annotation data (Gao et al., 2017), data on biochemical pathways or other data on biological mechanisms, or general (quantitative genetics) knowledge on -for instance- ratios of variances (Hem et al., 2021). Such approaches have already been followed in general genomic prediction literature, but we think that they will especially unfold their potential in the context of genetic resources.

A promising approach to follow for a broader range of traits is the comparison of structure, function and point of action of gene products. Given that some genes involved in the variation of stress resilience are known, bioinformatics tools can identify related genes whose gene products are of similar structure, have a similar predicted function or are relevant in the same biochemical pathways as the known genes. Genomic data can then be used to identify novel variation in the regions around these newly identified genes. Approaches of this kind have been used, for instance as resistance gene enrichment sequencing targeting certain protein motifs to identify resistances to biotic stresses (Jupe et al., 2013; Zhang et al., 2020), and have produced impressive results. However, such a strategy focuses on major gene effects and it remains to be seen whether they can be transferred to a quantitative trait such as yield under abiotic stress.

For the identification of germplasm bank accessions providing beneficial alleles for quantitative traits, we see the accession passport data as central information. This data cannot only be used to identify major effects in an association study, but can also be used in a genomic prediction approach. Here, a genomic relationship matrix of the accessions can be used to predict the environmental variables of the collection sites as “quantitative trait.” This “environmental genomic prediction” (EnvGP) then employs the environmental data as a phenotype in the training panel to predict materials of higher value for “hands-on” evaluation. Considering the polygenic nature of many traits of interest, we are currently assessing the potential of EnvGP together with other paradigms such as crop modeling to leverage genetic resources for germplasm development.

As an example addressing the process of repackaging of polygenic variation into elite and adapted materials, we cite Origin Specific Genomic Selection (OSGS; Yang et al., 2020). Here, the additional information used in the prediction is only the knowledge from which parent the alleles are derived. However, this add-on allows a partitioned form of genomic selection which facilitates a more targeted management of the introgression of novel beneficial variation during the introgression process. The genetic value is split into the contribution of the elite parent and the contribution of the “exotic” parent. Having both parts separated, the approach aims at avoiding a systematic selection against exotic alleles due to the higher genetic value of elite material although a certain fraction of exotic alleles may be beneficial. Validation of this approach using simulation and application in existing barley and maize datasets suggests potential for use in polygenic trait introgression in bi- and potentially multi-parental populations.

## Conclusion

Germplasm bank accessions can be considered as crop “genetic insurance” for the genetic adaptation to increased abiotic and biotic stresses, in particular caused by climate change. As for other fields, “big data,” here describing the germplasm bank collections, needs innovative approaches for “data mining,” to identify and harness useful variation, and unleash its potential. We see a conceptual key in combining statistical prediction methods with additional data other than genotypes and phenotypes. Approaches of this type have been followed in genomic prediction literature, but we consider them as particularly promising when applied in the context of harnessing genetic resources. The type of data to use, and how to use it provide a large playground for the exploration of creative approaches.

## Author Contributions

JM wrote the first draft and managed the edits from other authors. All authors discussed and outlined the content of the opinion and approved the published version for publication.

## Conflict of Interest

The authors declare that the research was conducted in the absence of any commercial or financial relationships that could be construed as a potential conflict of interest.
